# *PD-L1* promoter methylation is a prognostic biomarker for biochemical recurrence-free survival in prostate cancer patients following radical prostatectomy

**DOI:** 10.18632/oncotarget.13161

**Published:** 2016-11-07

**Authors:** Heidrun Gevensleben, Emily Eva Holmes, Diane Goltz, Jörn Dietrich, Verena Sailer, Jörg Ellinger, Dimo Dietrich, Glen Kristiansen

**Affiliations:** ^1^ Institute of Pathology, University Hospital Bonn, Bonn, Germany; ^2^ Department of Otolaryngology, Head and Neck Surgery, University Hospital Bonn, Bonn, Germany; ^3^ Weill Cornell Medicine of Cornell University, Department of Pathology and Laboratory Medicine, New York, NY, USA; ^4^ Weill Cornell Medicine of Cornell University, Englander Institute for Precision Medicine, New York, NY, USA; ^5^ Department of Urology, University Hospital Bonn, Bonn, Germany

**Keywords:** PD-L1, prostate cancer, DNA methylation, prognostic biomarker

## Abstract

**Background:**

The rapid development of programmed death 1 (PD-1)/programmed death ligand 1 (PD-L1) inhibitors has generated an urgent need for biomarkers assisting the selection of patients eligible for therapy. The use of PD-L1 immunohistochemistry, which has been suggested as a predictive biomarker, however, is confounded by multiple unresolved issues. The aim of this study therefore was to quantify *PD-L1* DNA methylation (*mPD-L1*) in prostate tissue samples and to evaluate its potential as a biomarker in prostate cancer (PCa).

**Results:**

In the training cohort, normal tissue showed significantly lower levels of *mPD-L1* compared to tumor tissue. High *mPD-L1* in PCa was associated with biochemical recurrence (BCR) in univariate Cox proportional hazards (hazard ratio (HR)=2.60 [95%CI: 1.50-4.51], p=0.001) and Kaplan-Meier analyses (p<0.001). These results were corroborated in an independent validation cohort in univariate Cox (HR=1.24 [95%CI: 1.08-1.43], p=0.002) and Kaplan-Meier analyses (p=0.029). Although *mPD-L1* and PD-L1 protein expression did not correlate in the validation cohort, both parameters added significant prognostic information in bivariate Cox analysis (HR=1.22 [95%CI: 1.05-1.42], p=0.008 for *mPD-L1* and HR=2.58 [95%CI: 1.43-4.63], p=0.002 for PD-L1 protein expression).

**Methods:**

*mPD-L1* was analyzed in a training cohort from The Cancer Genome Atlas (n=498) and was subsequently measured in an independent validation cohort (n=299) by quantitative methylation-specific real-time PCR. All patients had undergone radical prostatectomy.

**Conclusions:**

*mPD-L1* is a promising biomarker for the risk stratification of PCa patients and might offer additional relevant prognostic information to the implemented clinical parameters, particularly in the setting of immune checkpoint inhibition.

## INTRODUCTION

Recently, the blockade of the programmed death 1 (PD-1)/programmed death ligand 1 (PD-L1) signaling pathway, either with antibodies against the receptor PD-1 (Nivolumab, Pembrolizumab, Pidilizumab) or its ligand PD-L1 (BMS-936559, MPDL3280A, MEDI4736, MSB001718C), has shown promising results in several advanced cancers, e.g. malignant melanoma, non-small cell lung cancer, bladder cancer, head and neck cancer, and renal cell cancer [1–2]. While the activation of the PD-1/PD-L1 pathway allows for the tumor to successfully elude the host's immune system *via* T-cell exhaustion, a therapy strategically targeting the PD-1/PD-L1 pathway seems to promote the immune response against the tumor [3–4]. Immunohistochemically detected PD-L1 expression has further been reported to be a predictive biomarker for the treatment with anti-PD-1/PD-L1 treatment [4].

We have previously shown that PD-L1 is highly expressed in aggressive primary prostate cancer (PCa) and is an independent predictor of biochemical disease progression [5]. Although clinical trials for the treatment of PCa so far have yielded conflicting results [2, 6], our findings indicate that PD-1/PD-L1 targeted therapy might be a novel treatment option for hormone-naive tumors. Due to interlaboratory and interobserver variation, however, the general reproducibility of immunohistochemical methods still remains challenging [7–8]. For the successful implementation of reliable biomarkers into clinical practice, the robust and reproducible quantification of DNA methylation instead of immunohistochemistry might be highly beneficial.

The epigenetic mechanism of DNA methylation plays a key role in several fundamental biological processes, e.g. development, cell differentiation, and gene silencing [9–10]. Furthermore, DNA methylation is often deregulated in human malignancies [11–14] suggesting that aberrantly methylated loci might be a valuable source for biomarkers [15–16]. As DNA methylation can also be robustly quantified in specimens with limited DNA abundance or formalin-fixed paraffin-embedded tissue (FFPET), in which DNA is highly degraded, methylation-based biomarkers prove to be promising diagnostic tools for clinical routine [17]. So far, various DNA methylation biomarkers have been successfully applied in a clinical setting [18]. The short stature homeobox 2 (*SHOX2*) and septin 9 (*SEPT9*), for instance, are employed as diagnostic and screening tools for the detection of lung cancer and colorectal cancer [19–20]. In addition, promoter methylation of the O-6-methylguanine-DNA methyltransferase (*MGMT*) has been shown to be predictive of response to alkylating agents in glioblastoma [21–22].

We have very recently shown that promoter methylation of the immune checkpoint receptor PD-1 is an independent prognostic biomarker for biochemical recurrence (BCR)-free survival in PCa patients following radical prostatectomy [23]. The aim of the present study was to quantify *PD-L1* DNA methylation in prostate tissue samples and to evaluate its potential role as a prognostic biomarker in PCa.

## RESULTS

### PD-L1 promoter methylation and mRNA expression in prostate cancer patients (training cohort)

**Figure 1:**
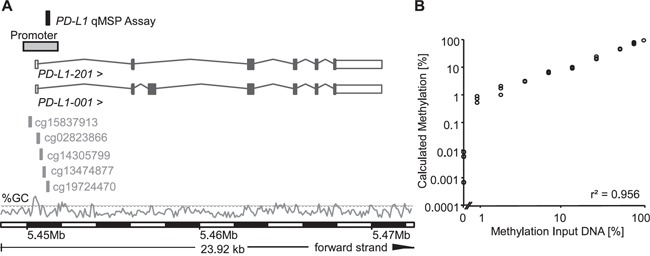
Genomic location, design, and validation of the *PD-L1* qPCR assay **A.** The *PD-L1* qMSP assay was located on the forward strand of chromosome 9. Both *PD-L1* spice variants *PD-L1-001* and *-201* are shown. The five cg-beads from the Illumina Infinium HumanMethylation450 BeadChip (cg15837913, cg02823866, cg14305799, cg13474877, and cg19724470) used for *PD-L1* methylation in the TCGA dataset are indicated. Information is based on Ensembl Homo sapiens version 82.37 (GRCh37.p3). The GC content [%] is shown with the dashed line indicating 50% GC. **B.** The quantitative real-time PCR assay was validated on a dilution series of bisulfite-converted artificially methylated and unmethylated DNA using the *PD-L1*/*ACTB* qMSP assay. Each sample was measured in triplicate.

**Figure 2:**
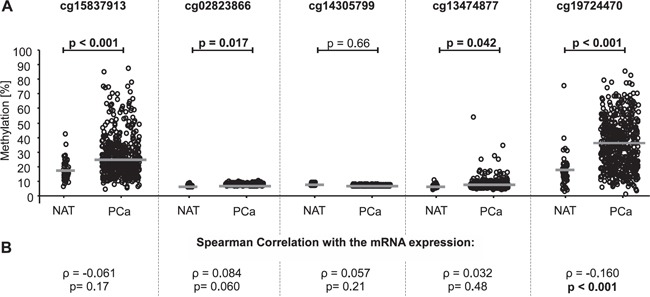
*PD-L1* DNA methylation in normal adjacent tissue (NAT) compared to prostate cancer (PCa) tissue and correlation of *PD-L1* methylation with mRNA expression in the training cohort **A.** Data for five beads from the Illumina Infinium HumanMethylation450 BeadChip located in the *PD-L1* gene. The median methylation is indicated by the grey line. P-values refer to Kruskal-Wallis test. *PD-L1* DNA methylation was shown to be significantly higher in PCa tissue compared to NAT in four out of five beads (cg15837913, cg02823866, cg13474877, and cg19724470). **B.** The correlation between mRNA expression and *PD-L1* DNA methylation was analyzed by Spearman's rank correlation showing a significant association of *PD-L1* methylation and mRNA expression for bead cg19724470.

For the analysis of *PD-L1* promoter methylation (*mPD-L1)* in the training cohort, five Illumina Infinium HumanMethylation450 BeadChip beads (cg15837913, cg02823866, cg14305799, cg13474877, and cg19724470) located in the promoter region of the *PD-L1* gene were used (Figure 1A). The results from the training cohort are entirely based upon data generated by The Cancer Genome Atlas (TCGA) Research Network: http://cancergenome.nih.gov/. Firstly, *mPD-L1* was analyzed in PCa (n = 498) and normal adjacent tissue (NAT, n = 65) samples from the training cohort. According to four of the five beads (cg15837913, cg02823866, cg13474877, and cg19724470), normal patient tissue showed significantly lower levels of *mPD-L1* compared to tumor tissue (Figure 2A). One bead (cg19724470) further showed a significant inverse correlation with PD-L1 mRNA expression (ρ = −0.160, p < 0.001, Figure 2B and Table 1). PD-L1 mRNA expression itself did not add prognostic value in Cox proportional hazards analysis (hazard ratio (HR) = 0.98 [95%CI: 0.95-1.02], p = 0.39).

**Table 1:** Association and correlation of *PD-L1* DNA methylation with mRNA expression and BCR-free survival

Bead	Correlation with mRNA ( ρ, p-value)	Cox (HR, [95%CI], p-value)^†^	Optimized cut-off [%]	Cox (HR, [95%CI], p-value)
cg15837913	−0.061, 0.17	1.01 [95%CI: 0.99-1.03], p = 0.29	35.845	2.07 [95%CI: 1.19-3.59], p = 0.010
cg02823866	0.084, 0.060	0.97 [95%CI: 0.61-1.54], p = 0.89	3.67	1.12 [95%CI: 0.51-2.47], p = 0.79
cg40305799	0.057, 0.21	0.78 [95%CI: 0.31-1.96], p = 0.59	1.764	0.68 [95%CI: 0.40-1.16], p = 0.15
cg13474877	0.032, 0.48	0.96 [95%CI: 0.87-1.07], p = 0.47	4.583	0.68 [95%CI: 0.32-1.45], p = 0.32
cg19724470	−0.160, <0.001	1.02 [95%CI: 1.00-1.04], p = 0.024	52.75	2.60 [95%CI: 1.50-4.51], p = 0.001

† *mPD-L1* analyzed as continuous variable

**Figure 3:**
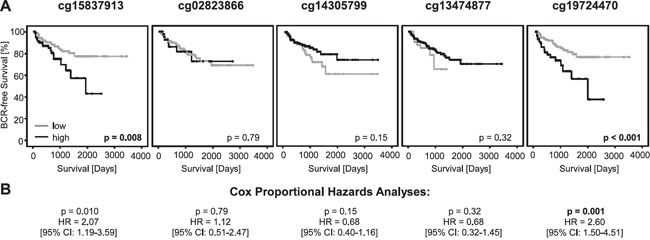
Kaplan-Meier analysis of *PD-L1* DNA methylation in prostate cancer patients (training cohort) **A.** Data for five beads from the Illumina Infinium Human Methylation 450 Bead Chip located in the *PD-L1* Gene. Patient samples were stratified into *mPD-L1*_low_ (grey) and *mPD-L1*_high_ (black) using an optimized cut-off. Kaplan-Meier analysis of *PD-L1* DNA methylation showed a better prognosis for the *mPD-L1*_low_ subgroup according to the bead pairs cg15837913 and cg19724470. **B.** Univariate Cox proportional hazard analyses conducted using an optimized cut-off corroborated the prognostic value of *mPD-L1*.

*mPD-L1* as continuous variable was further shown to be of prognostic significance in univariate Cox proportional hazard analysis for bead cg19724470 (HR = 1.02 [95%CI: 1.00-1.04], p = 0.024; Table 1). In order to analyze the suitability of *mPD-L1* for the stratification of patients at risk for BCR, *mPD-L1* was dichotomized using an optimized cut-off which was identified by an iterative approach. According to two beads (cg19724470 and cg15837913), *mPD-L1* was significantly associated with BCR in Kaplan-Meier analysis (cg19724470: p < 0.001, cg15837913: p = 0.008, Figure 3A) and the univariate Cox proportional hazards model (cg19724470: HR = 2.60 [95%CI: 1.50-4.51], p = 0.001; cg15837913: HR = 2.07 [95%CI: 1.19-3.59], p = 0.010, Table 1 and Figure 3B).

A detailed analysis of the association/correlation of *PD-L1* promoter methylation with clinicopathological parameters was further conducted by means of the best performing bead cg19724470. Hereby, *PD-L1* promoter methylation was shown to correlate/associate with the prognostic variables age (p = 0.028), pathological tumor category (pT) (p < 0.001), the International Society of Urological Pathology (ISUP) grading group (p = 0.001), surgical margin (p = 0.013), and ETS-related gene (ERG) expression (p = 0.022, Table 2).

**Table 2:** Patients' characteristics and correlation/association of *PD-L1* DNA promoter methylation (*mPD-L1*) with clinicopathological parameters in the training (n = 498, Probe cg19724470) and validation cohort (n = 299). *mPD-L1* was dichotomized according to the respective optimized cut-off (*mPD-L1*_low_ vs. *mPD-L1*_high_)

	Training Cohort								Validation Cohort							
	All patients	[%]	Median *mPD-L1* [%]	*mPD-L1*_low_	[%]	*mPD-L1*_high_	[%]	p-value	All patients	[%]	Median *mPD-L1* [%]	*mPD-L1*_low_	[%]	*mPD-L1*_high_	[%]	p-value
**Patients [n]**	498	100.0	36.3	397	79.7	101	20.3		299	100.0	0.49	197	65.9	102	34.1	
Patients with follow-up [n]	410															
Mean follow-up [months]	22								66							
Median follow-up [months]	16								63							
**Age [years]**																
Mean	61															
Median	61															
≤60	224	45.0	33.6	184	36.9	40	8.0		75	25.1	0.42	60	20.1	15	5.0	
>60	274	55.0	38.0	213	42.8	61	12.2		223	74.6	0.54	136	45.5	87	29.1	
Unknown	0	0.0						0.028*	1	0.3						0.10*
**Tumor category**																
pT2	188	37.8	32.0	164	32.9	24	4.8		205	68.6	0.41	147	49.2	58	19.4	
pT3	303	60.8	40.5	228	45.8	75	15.1		94	31.4	0.80	50	16.7	44	14.7	
Unknown	7	1.4						<0.001*	1	0.3						0.010*
**ISUP grading group (Gleason)**																
1 (<7)	45	9.0	27.4	40	8.0	5	1.0		166	55.5	0.34	123	41.1	43	14.4	
2 (3+4)	147	29.5	33.4	127	25.5	20	4.0		54	18.1	0.85	29	9.7	25	8.4	
3 (4+3)	101	20.3	37.4	84	16.9	17	3.4		24	8.0	0.35	17	5.7	7	2.3	
4 (=8)	64	12.9	34.9	46	9.2	18	3.6		37	12.4	0.82	21	7.0	16	5.4	
5 (>8)	141	28.3	41.2	100	20.1	41	8.2		16	5.4	1.52	5	1.7	11	3.7	
Unknown	0	0.0						**0.001**^†^	2	0.7						**0.001**^†^
**Surgical margin**																
R0	316	63.5	33.8	267	53.6	49	9.8		197	65.9	0.47	132	44.1	65	22.7	
R1	152	30.5	40.7	109	21.9	43	8.6		98	32.8	0.61	61	20.4	37	12.4	
Unknown	30	6.0						0.013*	4	1.3						0.14*
**Nodal category**																
pN0	346	69.5	36.5	274	55.0	72	14.5		278	93.0	0.49	185	61.9	93	31.1	
pN1	79	15.9	36.4	61	12.2	18	3.6		20	6.7	0.48	11	3.7	9	3.0	
Unknown	73	14.7						0.88*	1	0.3						0.90*
**Pre-surgical PSA [ng/ml]**																
0-4	53	10.6	29.8	48	9.6	5	1.0		27	9.0	0.77	16	5.4	11	3.7	
4-10	286	57.4	35.2	229	46.0	57	11.4		173	57.9	0.42	121	40.5	52	17.4	
>10	156	31.3	40.5	118	23.7	38	7.6		86	28.8	0.61	52	17.4	34	11.4	
Unknown	3	0.6						0.11^†^	13	4.3						0.72^†^
**ERG^#^**																
Negative	178	35.7	34.4	146	29.3	32	6.4		145	48.5	0.57	94	31.4	51	17.1	
Positive	152	30.5	40.6	121	24.3	31	6.2		65	21.7	0.88	35	11.7	30	10.0	
Unknown	168	33.7						0.022*	89	29.8						0.19*
**AR score^§^**																
Negative	166	33.3	36.6	136	27.3	30	6.0		83	27.8	0.33	54	18.1	29	9.7	
Positive	167	33.5	37.7	134	26.9	33	6.6		81	27.1	0.71	54	18.1	27	9.0	
Unknown	165	33.1						0.79*	136	45.5						0.24*

* Mann-Whitney U test (Wilcoxon Rank-sum test)

† Kruskal-Wallis test

# Training cohort: ERG fusion (as adopted from [34]); validation cohort: nuclear ERG protein expression (as previously published in [35])

§ Training cohort: AR activity score (as adopted from [34]); validation cohort: AR protein expression (as previously published in [35])

### Design and validation of a PD-L1 qMSP assay

To further evaluate the previous results in an independent dataset with an additional technology, a quantitative methylation-specific real-time PCR (qMSP) assay was designed within the promoter region of the *PD-L1* gene in the location of the best performing cg19724470 bead (Figure 1A). In brief, the assay represents a duplex real-time PCR for sensitive and quantitative detection of *PD-L1* DNA methylation and a reference PCR for quantification of the total DNA using the *ACTB* locus. The assay performance was validated using a dilution series of bisulfite-converted artificially methylated and unmethylated DNA. Samples were measured in triplicate. As indicated in Figure 1B, the *PD-L1/ACTB* qMSP assay allowed for a highly accurate quantification of the *PD-L1* DNA methylation over a broad range of relative methylation of the template DNA, even at low methylation levels. The assay showed an accurate quantification of *mPD-L1* within the whole spectrum from 0% to 100% methylation (r^2^ = 0.956, Figure 1B).

### PD-L1 promoter methylation in prostate cancer patients (validation cohort)

**Figure 4:**
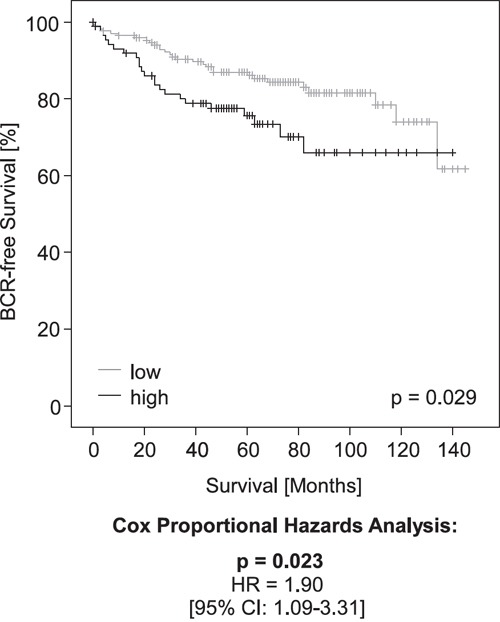
Kaplan-Meier analysis of *PD-L1* DNA methylation in prostate cancer patients (validation cohort) Patient samples were stratified into *mPD-L1*_low_ (grey) and *mPD-L1*_high_ (black) according an optimized cut-off. Kaplan-Meier analysis of *PD-L1* DNA methylation showed a better prognosis for the *mPD-L1*_low_ patient subgroup. Univariate Cox proportional hazard analyses conducted using an optimized cut-off corroborated the prognostic value of *mPD-L1*.

In a validation cohort of 299 patients with clinical follow-up, *PD-L1* DNA methylation dichotomized by an optimized cut-off (*mPD-L1*_low_ < 0.98% ≤ *mPD-L1*_high_) significantly associated with prognostic clinicopathological variables including pT category (p = 0.010), and ISUP grading group (p = 0.001, Table 2). Furthermore, high *PD-L1* DNA methylation was significantly associated with an increased risk for BCR in patients. In univariate Cox proportional hazards analysis, *mPD-L1* was significantly associated with BCR when analyzed as a continuous variable (HR = 1.24 [95%CI: 1.08-1.43], p = 0.002) and as a dichtomized variable using an optimized cut-off (HR = 1.90 [95%CI: 1.09-3.31], p = 0.023, Table 3). The prognostic value of *mPD-L1* was further confirmed by Kaplan-Meier analysis (p = 0.029 for optimized cut-off, Figure 4).

**Table 3:** Univariate Cox proportional hazards analysis on biochemical recurrence-free survival in the training (n = 410, Probe cg19724470) and validation cohort (n = 259) of prostate cancer patients treated by radical prostatectomy

	Univariate Cox Analysis			
	Training Cohort		Validation Cohort	
	Hazard ratio [95% CI]	p-value	Hazard ratio [95% CI]	p-value
pT category (pT3 & pT4 vs. pT2 & pT1)	5.37 [2.14-13.5]	**<0.001**	2.70 [1.56-4.69]	**<0.001**
ISUP grading group (1 vs. 2 vs. 3 vs. 4 vs. 5)	1.69 [1.34-2.13]	**<0.001**	2.00 [1.65-2.44]	**<0.001**
Surgical margin (R1 vs. R0)	1.49 [0.87-2.56]	0.15	2.31 [1.32-4.06]	**0.003**
Nodal status (pN1 vs. pN0)	1.84 [1.00-3.35]	**0.049**	1.39 [0.55-3.50]	0.49
Preoperative PSA level (continuous)	1.04 [1.02-1.05]	**<0.001**	1.01 [1.00-1.02]	0.11
AR expression^§^ (AR positive vs. AR negative)	0.96 [0.49-1.89]	0.91	0.76 [0.50-1.16]	0.20
ERG^#^ (ERG-positive vs. ERG-negative)	0.80 [0.40-1.57]	0.51	0.77 [0.40-1.50]	0.44
Age (continuous)	1.02 [0.98-1.06]	0.39	1.01 [0.97-1.06]	0.56
*mPD-L1* (continuous)	1.02 [1.00-1.03]	**0.024**	1.24 [1.08-1.43]	**0.002**
*mPD-L1* (optimized cut-off, *mPD-L1_high_* vs. *mPD-L1_low_*)	2.60 [1.50-4.51]	**0.001**	1.90 [1.09-3.31]	**0.023**

# Training cohort: ERG fusion (as adopted from [34]); validation cohort: nuclear ERG protein expression (as previously published in [35])

§ Training cohort: AR activity score (as adopted from [34]); validation cohort: AR protein expression (as previously published in [35])

### Association of PD-L1 promoter methylation and protein expression (validation cohort)

**Figure 5:**
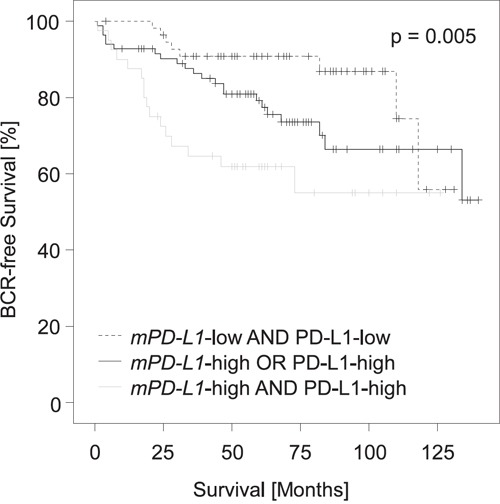
Kaplan-Meier analysis of *PD-L1* DNA methylation in combination with PD-L1 protein expression in prostate cancer patients (validation cohort) Patient samples (n = 209) were stratified according to *mPD-L1* status (high *vs.* low) in combination with dichotomized PD-L1 protein expression status as determined earlier [5]. Patients with high PD-L1 protein expression and high *mPD-L1* had shorter BCR-free intervals compared to patients with simultaneous low PD-L1 protein expression and low *mPD-L1*. Patients with either high PD-L1 protein expression and low *mPD-L1* or low PD-L1 protein expression and high *mPD-L1* showed intermediate BCR-free intervals.

High PD-L1 protein expression determined semi-quantitatively *via* immunohistochemistry (IHC) has earlier been shown to be a strong predictor of BCR in the cohort under investigation (p = 0.004, HR = 2.37 [95%CI: 1.32-4.25]) [5]. The fact that high *mPD-L1* and high PD-L1 protein expression have an adverse prognostic value in the validation cohort seems to be conflicting, since DNA methylation is regarded as a long-lasting negative regulator of mRNA expression and should thus be associated with silencing of the gene product. We therefore analyzed the correlation between DNA methylation and protein expression. Matched IHC and DNA methylation results were available for n = 209 patients. Our results suggested that PD-L1 protein expression is unrelated to *mPD-L1* in the PCa specimens available for comparative examination (ρ = 0.05, p = 0.47), indicating that post-transcriptional and potentially post-translational regulatory mechanisms are involved in the control of PD-L1 protein expression. In order to test whether *mPD-L1* and PD-L1 protein expression provide independent prognostic information, we performed a multivariate Cox proportional hazards analysis including both variables. Notably, *PD-L1* methylation (HR = 1.22 [95%CI: 1.05-1.42] p = 0.008) and PD-L1 protein expression (HR = 2.58 [95%CI: 1.43-4.63], p = 0.002) analyzed as continuous variables both added significant prognostic information. We further analyzed the prognosis of PCa patients stratified according to their *mPD-L1* status (high *vs.* low) in combination with the dichotomized PD-L1 protein expression status. Figure 5 shows that patients with high PD-L1 protein expression and high *mPD-L1* present with shorter BCR-free intervals compared to patients with simultaneous low PD-L1 protein expression and low *mPD-L1*. Patients with either high PD-L1 protein expression and low *mPD-L1* or low PD-L1 protein expression and high *mPD-L1* showed an intermediate BCR-free interval. The finding that PD-L1 protein expression and methylation seem to be unrelated in our cohort prompted us to investigate additional mechanisms of post-transcriptional regulation of PD-L1.

### Association of PD-L1 mRNA and methylation with microRNA expression (training cohort)

Known epigenetic post-translational modification mechanisms of PD-L1 include the differential expression of microRNA (miR). In particular miR-197, miR-200, miR-570, miR-34a, and miR-513 have been shown to play a key role in the regulation of PD-L1 expression (reviewed by [24]). MiRs are non-coding single-stranded RNAs 22–24 nucleotides in length, which can bind and degrade or stabilize mRNA. Data on miR expression were available only for the training cohort. MiR-197 and miR-200a-c positively correlated with PD-L1 mRNA and inversely correlated with *mPD-L1*, suggesting that *mPD-L1* was associated not only with decreased mRNA expression but also with lower levels of mRNA destabilizing miR-197 and three of the miR-200 family members miR-200a-c (Table 4). MiR-570, on the other hand, was only correlated with *mPD-L1.* For miR-34a, an inverse correlation with *mPD-L1* and mRNA expression was shown. MiR-513 was not differentially expressed with regard to methylation and mRNA expression of PD-L1.

**Table 4:** Correlation of microRNA (miR) expression (miR-197, miR-200, miR-570, miR-34a, and miR-513) with *PD-L1* methylation and mRNA expression levels

			*mPD-L1*	PD-L1 mRNA
Spearman's rho (ρ)	mPD-L1	ρ	1.000	**-0.160**
		*p*-value		**<0.001**
		n	498	497
	miR-197	ρ	**0.147**	**-0.112**
		*p*-value	**0.001**	**0.013**
		n	494	493
	miR-200a	ρ	**0.121**	**-0.140**
		*p*-value	**0.007**	**0.021**
		n	494	493
	miR-200b	ρ	**0.136**	**-0.139**
		*p*-value	**0.003**	**0.002**
		n	494	493
	miR-200c	ρ	**0.273**	**-0.258**
		*p*-value	**<0.001**	**<0.001**
		n	494	493
	miR-34a	ρ	**-0.132**	**-0.141**
		*p*-value	**0.003**	**0.002**
		n	494	493
	miR-513a_1_	ρ	−0.044	0.009
		*p*-value	0.33	0.84
		n	494	493
	miR-513a_2_	ρ	−0.021	−0.035
		*p*-value	0.64	0.44
		n	494	493
	miR-513b	ρ	−0.031	0.020
		*p*-value	0.50	0.66
		n	494	493
	miR-513c	ρ	−0.072	.070
		*p*-value	0.11	0.12
		n	494	493
	miR-570	ρ	0.038	**-0.123**
		*p*-value	0.39	**0.006**
		n	494	493

## DISCUSSION

Targeting the immune checkpoint pathway PD-1/PD-1 has emerged as a promising therapy providing significant clinical activity and durable benefits with minimal toxicities in several solid tumors [1–2]. The rapid development and approval of PD-1/PD-L1 inhibitors has generated an urgent need for predictive biomarkers assisting the selection of patients most likely to respond to therapy. Several clinical trials have suggested immunohistochemically detected PD-L1 expression as a predictive biomarker for the treatment with anti-PD-1/PD-L1 inhibitors [4, 25]. The use of PD-L1 immunohistochemistry as a predictive biomarker, however, is confounded by multiple unresolved issues e.g. differing antibodies and scoring cut-offs, tissue preparation, as well as interlaboratory and interobserver variability.

Aberrant DNA methylation of certain loci has been reported to play a major role in carcinogenesis [26]. Such epigenetic modifications have further been shown to be a robust and reliable diagnostic tool [19, 21–22, 27], therefore representing a promising source for cancer biomarkers. We have very recently shown that promoter methylation of the immune checkpoint receptor PD-1 is an independent prognostic biomarker for biochemical recurrence (BCR)-free survival in PCa patients following radical prostatectomy [23]. So far, no data are available on the role of *PD-L1* promoter methylation in PCa. The aim of this study therefore was to quantify *PD-L1* DNA methylation in prostate tissue samples and to evaluate its potential as a biomarker in PCa.

DNA methylation of promoter regions is often associated with epigenetic silencing [12]. In normal tissue around 80% of CpGs are methylated and mainly CpG islands in promoter regions of active genes are hypomethylated [28]. In cancer there is a shift in the pattern of DNA methylation towards a global hypomethylation, whereas certain CpGs, often in promoter regions of tumor suppressor genes, are thought to become hypermethylated [28–29]. Nevertheless, recent publications show that the aberrant DNA methylation, especially in cancer, is much more complex (reviewed by [12]).

In the present study, five Illumina Infinium HumanMethylation450 BeadChip beads taken from the TCGA dataset and located in the promoter region of the *PD-L1* gene were analyzed. In this training cohort, *mPD-L1* levels were increased significantly in PCa compared to normal tissue, indicating that *PD-L1* might be differentially methylated in prostatic malignancies. Further investigations revealed that two of the five examined beads (cg19724470 and cg15837913) showed a significant correlation of *mPD-L1* with BCR in univariate Cox proportional hazards analysis and Kaplan-Meier analysis. Interestingly, differential methylation was found in the border regions and not the centre of the CpG-dense promoter area. One of the two beads (cg19724470) further showed an inverse correlation with PD-L1 mRNA expression and an association with prognostic clinicopathological variables including the ISUP grading group, pT, pre-surgical prostate-specific antigen (PSA), resection margin, and ERG status.

In order to validate these findings with an additional technology in an independent cohort, a quantitative methylation qPCR assay was designed within the region of the best performing bead (cg19724470). In this validation cohort, high *PD-L1* DNA methylation, analyzed as a continuous variable and as a dichtomized variable using an optimized cut-off, was significantly associated with an increased risk for BCR in patients in univariate Cox proportional hazards analysis and its prognostic value was further confirmed by Kaplan-Meier analysis.

Surprisingly, our study revealed no inverse correlation of *PD-L1* promoter methylation and protein expression in PCa. This may point at a complex post-transcriptional and post-translational regulation of PD-L1. According to our findings, *mPD-L1* is inversely correlated with mRNA transcription and associated with miR as cellular component, which modify the downstream processing of PD-L1 mRNA. Differential expression of miR may therefore potentially interfere with the linear translation of PD-L1 mRNA into PD-L1 protein. Thus, further studies are warranted to unravel the complex interplay between DNA methylation and protein expression in PCa. In addition, post-translational modifications which affect the stability of the protein (e.g. glycolization) and in turn alter the affinity of the anti-PD-L1 antibody to membranous PD-L1 might further be causative for ostensibly conflicting results [30]. When combining PD-L1 protein expression and methylation, however, both parameters added independent prognostic information, suggesting that PD-L1 methylation might be a valuable companion biomarker in addition to immunohistochemistry. We would therefore strongly recommend the integration of its analysis in running clinical trials.

A limitation of DNA methylation analysis techniques that needs to be mentioned is the inability to differentiate heterogeneous methylation patterns in different cell types present within samples. Methylation analysis is performed using tissue lysates and not specific cell subtypes, which can differ in methylation. We have previously shown that methylation levels in breast cancer differ among ductal epithelia of adjacent normal tissue, stromal cells, tumor infiltrating lymphocytes, and even within the invasive tumor itself [31]. Further studies employing microdissected specimens are therefore warranted to analyze tissue- and cell-specific epigenetic regulation in detail.

Taken together, our findings might be of great value for the tailoring of individual therapies and risk stratification, especially within the framework of therapies targeting the PD-1/PD-L1 signaling pathway. Although the experience with PD-1/PD-L1 inhibition in PCa is still limited [4], *PD-1* DNA methylation may have a predictive value for future treatment with checkpoint inhibitors in PCa. However, further studies are warranted to determine the potential of *PD-L1* methylation as a biomarker, particularly in the setting of immune checkpoint inhibition.

## MATERIALS AND METHODS

### Patients and tissue samples

#### Training cohort

The patient cohort based on data collected by The Cancer Genome Atlas (TCGA) comprised of 498 patients with histologically confirmed PCa obtained from several international centers involved in the TCGA project [32]. BCR-free survival was considered as the primary endpoint of the study. Informed consent was acquired from all patients included in the cohort in accordance with the Helsinki Declaration of 1975. PD-L1 mRNA expression data were available for 497 patients samples. *PD-L1* promoter methylation was assessable for 498 specimens. Clinical follow-up was available for 410 individuals (mean follow-up period 21.6 months, range 1-133 months). Data on miR expression were obtainable for 494 patients.

#### Validation cohort

A total of 299 patients with histologically confirmed PCa who had undergone radical prostatectomy at the University Hospital Bonn between 1998 and 2008 were included in the study. BCR-free survival defined as postoperative PSA levels rising above 0.2 ng/ml were determined as the primary endpoint of the study. Clinical follow-up was available for 259 individuals (mean follow-up period 66.4 months, range 1-145 months).

### Ethical approval

The section of the study including patient material from the University Hospital Bonn has been approved by the local Institutional Review Board at the University Hospital Bonn (Board number 071/14), which waived the need for written informed consent from the participants. All experiments were performed in accordance with the relevant guidelines and regulations.

### Sample preparation and bisulfite conversion

For methylation analysis, FFPET samples were processed according to the InnuCONVERT Bisulfite All-In-One Kit (Analytik Jena) as previously published [33]. For assay validation, a dilution series of bisulfite-converted, unmethylated sperm DNA (NW Andrology & Cryobank Inc., Spokane, WA, USA) and artificially methylated DNA (CpGenome™ Universal Methylated DNA; Merck Millipore, Darmstadt, Germany) and was used. DNA concentration was quantified by UV spectrophotometry using a Nanodrop ND-1000 spectral photometer (Nanodrop Technologies, Wilmington, DE, USA).

### PD-L1 quantitative methylation real-time PCR

The DNA methylation of *PD-L1* was determined using a qMSP assay. Primers and probes are shown in Table 5. Relative DNA methylation of the *PD-L1* locus compared to total DNA (determined via ACTB reference in a duplex PCR reaction) was quantified using the AB 7500 Fast Real-Time PCR System (Life Technologies Corporation, Carlsbad, CA, USA). Each patient sample was measured in triplicate and an input of 50ng bisulfite-converted DNA per reaction was measured. Thresholds and baselines were set as follows: 0.07 (threshold *PD-L1*), 3-22 (baseline PD-L1), 0.02 (threshold *ACTB*), and 3-21 (baseline ACTB). Percentage DNA methylation was calculated using the following formula: ΔCT = CT_PD-L1_ – CT_ACTB_, ΔΔCT = ΔCT_Sample_ - ΔCT_Calibrator_, Methylation[%]=100%*2^ΔΔCT^

**Table 5:** Primer and probe sequences used for the *PD-L1* methylation-specific qPCRs

Primer/Probe	Sequence
*PD-L1* forward primer	5′-ATATAAAATAAATAATCATTCTTATACG-3′
*PD-L1* reverse primer	5′-CGTTTAGGGATTTTGGATTTGTTTAGC-3′
*PD-L1* detection probe	5′-FAM-CACGAATCCAAATCCACCGCCAAC-BHQ1-3′
*ACTB* forward primer	5′-GTGATGGAGGAGGTTTAGTAAGTT-3′
*ACTB* reverse primer	5′-CCAATAAAACCTACTCCTCCCTTAA-3′
*ACTB* detection probe	5′-Atto 647N-ACCACCACCCAACACACAATAACAAACACA-BHQ1-3′

### Statistical analysis

The statistical analyses were performed using SPSS, version 22 (SPSS Inc., Chicago, IL). Associations/correlations between *PD-L1* methylation, expression, and clinicopathological variables were analyzed using the Spearman's rank correlation, Kruskal-Wallis (≥ 3 groups) or Mann-Whitney-U (2 groups) tests. BCR-free survival analyses were conducted by Kaplan-Meier and Cox proportional hazards regression analyses. P-values < 0.05 were considered statistically significant.

## Abbreviations

BCR: Biochemical recurrence
ERG: ETS-related gene
FFPET: Formalin-fixed paraffin-embedded tissue
HR: Hazard ratio
ISUP: International Society of Urological Pathology
*MGMT*: O^6^-methylguanine-DNA methyltransferase
*mPD-L1*: PD-L1 promoter methylation
pT: Pathological tumor category
NAT: Normal adjacent tissue
PCa: Prostate cancer
PD-1: Programmed death 1
PD-L1: Programmed death ligand 1
PSA: Prostate-specific antigen
qMSP: Quantitative methylation-specific PCR
*SHOX2*: Short stature homeobox 2
*SEPT9*: Septin-9
TCGA: The Cancer Genome Atlas.

## References

[1] Hamid O, Robert C, Daud A, Hodi FS, Hwu WJ, Kefford R, Wolchok JD, Hersey P, Joseph RW, Weber JS, Dronca R, Gangadhar TC, Patnaik A, Zarour H, Joshua AM, Gergich K (2013). Safety and tumor responses with lambrolizumab (anti-PD-1) in melanoma. N Engl J Med.

[2] Sznol M, Chen L (2013). Antagonist antibodies to PD-1 and B7-H1 (PD-L1) in the treatment of advanced human cancer. Clin Cancer Res.

[3] Brahmer JR, Tykodi SS, Chow LQ, Hwu WJ, Topalian SL, Hwu P, Drake CG, Camacho LH, Kauh J, Odunsi K, Pitot HC, Hamid O, Bhatia S, Martins R, Eaton K, Chen S (2012). Safety and activity of anti-PD-L1 antibody in patients with advanced cancer. N Engl J Med.

[4] Topalian SL, Hodi FS, Brahmer JR, Gettinger SN, Smith DC, McDermott DF, Powderly JD, Carvajal RD, Sosman JA, Atkins MB, Leming PD, Spigel DR, Antonia SJ, Horn L, Drake CG, Pardoll DM (2012). Safety, activity, and immune correlates of anti-PD-1 antibody in cancer. N Engl J Med.

[5] Gevensleben H, Dietrich D, Golletz C, Steiner S, Jung M, Thiesler T, Majores M, Stein J, Uhl B, Mueller S, Ellinger J, Stephan C, Jung K, Brossart P, Kristiansen G (2015). The immune checkpoint regulator PD-L1 is highly expressed in aggressive primary prostate cancer. Clin Cancer Res.

[6] Slovin SF, Higano CS, Hamid O, Tejwani S, Harzstark A, Alumkal JJ, Scher HI, Chin K, Gagnier P, McHenry MB, Beer TM (2013). Ipilimumab alone or in combination with radiotherapy in metastatic castration-resistant prostate cancer: results from an open-label, multicenter phase I/II study. Ann Oncol.

[7] Parker RL, Huntsman DG, Lesack DW, Cupples JB, Grant DR, Akbari M, Gilks CB (2002). Assessment of interlaboratory variation in the immunohistochemical determination of estrogen receptor status using a breast cancer tissue microarray. Am J Clin Pathol.

[8] von Wasielewski R, Mengel M, Wiese B, Rudiger T, Muller-Hermelink HK, Kreipe H (2002). Tissue array technology for testing interlaboratory and interobserver reproducibility of immunohistochemical estrogen receptor analysis in a large multicenter trial. Am J Clin Pathol.

[9] De Carvalho DD, You JS, Jones PA (2010). DNA methylation and cellular reprogramming. Trends Cell Biol.

[10] Geiman TM, Muegge K (2010). DNA methylation in early development. Mol Reprod Dev.

[11] Baylin SB, Jones PA (2011). A decade of exploring the cancer epigenome - biological and translational implications. Nat Rev Cancer.

[12] Jones PA (2012). Functions of DNA methylation: islands, start sites, gene bodies and beyond. Nat Rev Genet.

[13] Shen H, Laird PW (2013). Interplay between the cancer genome and epigenome. Cell.

[14] Suva ML, Riggi N, Bernstein BE (2013). Epigenetic reprogramming in cancer. Science.

[15] Kulis M, Esteller M (2010). DNA methylation and cancer. Adv Genet.

[16] Sincic N, Herceg Z (2011). DNA methylation and cancer: ghosts and angels above the genes. Curr Opin Oncol.

[17] Dietrich D, Uhl B, Sailer V, Holmes EE, Jung M, Meller S, Kristiansen G (2013). Improved PCR performance using template DNA from formalin-fixed and paraffin-embedded tissues by overcoming PCR inhibition. PLoS One.

[18] Dietrich D, Meller S, Uhl B, Ralla B, Stephan C, Jung K, Ellinger J, Kristiansen G (2014). Nucleic acid-based tissue biomarkers of urologic malignancies. Crit Rev Clin Lab Sci.

[19] Church TR, Wandell M, Lofton-Day C, Mongin SJ, Burger M, Payne SR, Castanos-Velez E, Blumenstein BA, Rosch T, Osborn N, Snover D, Day RW, Ransohoff DF (2014). Prospective evaluation of methylated SEPT9 in plasma for detection of asymptomatic colorectal cancer. Gut.

[20] Dietrich D, Kneip C, Raji O, Liloglou T, Seegebarth A, Schlegel T, Flemming N, Rausch S, Distler J, Fleischhacker M, Schmidt B, Giles T, Walshaw M, Warburton C, Liebenberg V, Field JK (2012). Performance evaluation of the DNA methylation biomarker SHOX2 for the aid in diagnosis of lung cancer based on the analysis of bronchial aspirates. Int J Oncol.

[21] Esteller M, Garcia-Foncillas J, Andion E, Goodman SN, Hidalgo OF, Vanaclocha V, Baylin SB, Herman JG (2000). Inactivation of the DNA-repair gene MGMT and the clinical response of gliomas to alkylating agents. N Engl J Med.

[22] Wick W, Weller M, van den Bent M, Sanson M, Weiler M, von Deimling A, Plass C, Hegi M, Platten M, Reifenberger G (2014). MGMT testing-the challenges for biomarker-based glioma treatment. Nat Rev Neurol.

[23] Goltz D, Gevensleben H, Dietrich J, Ellinger J, Landsberg J, Kristiansen G, Dietrich D (2016). Promoter Methylation of the Immune Checkpoint Receptor PD-1 (PDCD1) Is an Independent Prognostic Biomarker for Biochemical Recurrence-free Survival in Prostate Cancer Patients Following Radical Prostatectomy. Oncoimmunology, Manuscript accepted.

[24] Chen J, Jiang CC, Jin L, Zhang XD (2016). Regulation of PD-L1: a novel role of pro-survival signalling in cancer. Ann Oncol.

[25] Motzer RJ, Rini BI, McDermott DF, Redman BG, Kuzel TM, Harrison MR, Vaishampayan UN, Drabkin HA, George S, Logan TF, Margolin KA, Plimack ER, Lambert AM, Waxman IM, Hammers HJ (2015). Nivolumab for Metastatic Renal Cell Carcinoma: Results of a Randomized Phase II Trial. J Clin Oncol.

[26] Ting AH, McGarvey KM, Baylin SB (2006). The cancer epigenome-components and functional correlates. Genes Dev.

[27] Dietrich D, Jung M, Puetzer S, Leisse A, Holmes EE, Meller S, Uhl B, Schatz P, Ivascu C, Kristiansen G (2013). Diagnostic and prognostic value of SHOX2 and SEPT9 DNA methylation and cytology in benign, paramalignant and malignant pleural effusions. PLoS One.

[28] Eckhardt F, Lewin J, Cortese R, Rakyan VK, Attwood J, Burger M, Burton J, Cox TV, Davies R, Down TA, Haefliger C, Horton R, Howe K, Jackson DK, Kunde J, Koenig C (2006). DNA methylation profiling of human chromosomes 6, 20 and 22. Nat Genet.

[29] Lee ST, Wiemels JL (2016). Genome-wide CpG island methylation and intergenic demethylation propensities vary among different tumor sites. Nucleic Acids Res.

[30] Li CW, Lim SO, Xia W, Lee HH, Chan LC, Kuo CW, Khoo KH, Chang SS, Cha JH, Kim T, Hsu JL, Wu Y, Hsu JM, Yamaguchi H, Ding Q, Wang Y (2016). Glycosylation and stabilization of programmed death ligand-1 suppresses T-cell activity. Nat Commun.

[31] Dietrich D, Lesche R, Tetzner R, Krispin M, Dietrich J, Haedicke W, Schuster M, Kristiansen G (2009). Analysis of DNA methylation of multiple genes in microdissected cells from formalin-fixed and paraffin-embedded tissues. J Histochem Cytochem.

[32] TCGA Cancer Genome Atlas http://cancergenomenihgov/.

[33] Holmes EE, Jung M, Meller S, Leisse A, Sailer V, Zech J, Mengdehl M, Garbe LA, Uhl B, Kristiansen G, Dietrich D (2014). Performance evaluation of kits for bisulfite-conversion of DNA from tissues, cell lines, FFPE tissues, aspirates, lavages, effusions, plasma, serum, and urine. PLoS One.

[34] (2015). The Molecular Taxonomy of Primary Prostate Cancer. Cell.

[35] Goltz D, Holmes EE, Gevensleben H, Sailer V, Dietrich J, Jung M, Roehler M, Meller S, Ellinger J, Kristiansen G, Dietrich D (2016). CXCL12 Promoter Methylation and PD-L1 Expression as Prognostic Biomarkers in Prostate Cancer Patients. Oncotarget.

